# Supplementary material for: Cytogenetic Assessment and Risk Stratification in Myelofibrosis with Optical Genome Mapping
Source: Cancers (Basel). 2023 Jun 2;15(11):3039. doi: 10.3390/cancers15113039 (PMC10252182; doi:10.3390/cancers15113039)
Supplement: Supplementary file 1 [file cancers-15-03039-s001.zip › cancers-2375272-supplementary.pdf]

Patient	UHMW DNA		Labeling		Run parameters							
	DNA concentration (ng/µl)	DNA CV	DNA concentration	DNA CV	Avg N50 (≥150kpb)	Avg N50 (≥20kpb)	Avg Label Density	Avg Map Rate	Effective Coverage	Avg PLV	Avg NLV	DNA collected
	**36-150**	**<0,3**	**4-12**	**<0,3**	**>230**	**>150**	**14-17 /100kbp**	**>70%**	**>300x**	**<10%**	**<15%**	**>1500 Gbp**
1	88,23	0,14	**12,80**	0,10	338	290	15,93	94,5	470,50	2,9	5,0	1537,29
2	103,70	0,14	9,17	0,01	297	260	15,66	93,7	466,79	3,4	5,3	1538,05
3	142,00	0,12	5,29	0,03	255	214	15,33	92,2	447,96	4,1	6,0	1500,20
4	115,67	0,10	7,39	0,07	292	244	16,78	89,9	450,02	3,3	5,2	1546,23
5	87,93	0,05	4,02	0,03	**214**	156	15,36	80,3	325,46	7,9	7,6	**1253,21**
6	119,40	0,23	4,37	0,09	**200**	**138**	15,25	77,7	**283,51**	7,7	7,8	**1128,00**
7	42,77	0,28	11,40	0,01	240	**131**	15,37	79,9	393,00	2,7	9,4	1522,86
8	76,53	0,04	4,27	0,00	**193**	**120**	16,57	**61,3**	**224,62**	5,0	7,2	**1148,68**
9	88,60	0,09	7,98	0,02	**220**	**117**	15,57	87,2	425,00	3,9	6,1	1505,00
10	104,63	0,09	6,84	0,01	**199**	**146**	15,65	86,4	420,83	3,2	5,5	1503,77
11	108,33	0,09	5,38	0,01	265	204	15,89	90,0	444,21	3,8	5,4	1523,00
12	106,67	0,04	7,34	0,01	274	228	16,00	92,9	463,56	4,3	5,0	1541,47
13	93,03	0,04	8,43	0,05	263	201	15,95	91,3	458,31	4,5	5,7	1549,97
14	134,33	0,05	4,85	0,01	245	202	16,68	87,0	423,00	5,5	5,8	1502,00
15	142,33	0,04	7,02	0,07	252	190	16,12	90,0	444,00	5,6	5,9	1524,00
16	114,67	0,08	4,71	0,00	261	202	15,40	90,0	444,00	5,1	5,6	1523,00
17	70,97	0,06	7,38	0,01	251	196	15,31	89,3	434,63	3,2	8,8	1502,84
18	84,80	0,11	10,03	0,01	321	256	18,44	71,7	350,39	3,5	14,5	1509,16
19	66,20	0,07	5,46	0,01	235	186	15,69	83,9	326,56	3,3	10,4	**1205,50**
20	40,80	0,07	10,60	0,01	229	182	14,84	87,1	423,63	1,9	12,7	1502,39
21	44,07	0,10	9,54	0,08	230	165	14,03	81,2	394,41	1,9	**17,1**	1500,43
Mean	88,71	0,08	6,94	0,03	248,42	186,11	15,78	85,19	399,20	3,86	7,18	1448,64
SD	31,23	0,03	2,31	0,01	76,37	88,39	1,34	9,40	53,80	0,71	8,56	26,06
Median	93,03	0,09	7,34	0,01	251	196	15,66	87,2	425	3,8	6	1505

The third row indicates the desired values according to the manufacturer's recommendations (Bionano Genomics).

Bold values indicate that they are out of range according to the protocol.

Abbreviations: UHMW DNA = Ultra-High Molecular Weight DNA; UHMW DNA concentration = average UHMW DNA concentration obtained after 3 measurements of the sample after a vortex of at least 30 seconds and quantified with Qubit dsDNA BR Assay; UHMW DNA CV = Coefficient of Variation (CV = standard deviation/mean) from the three readings; Labeled DNA concentration = average Labeled DNA concentration obtained after 2 measurements of the sample after a vortex of at least 30 seconds and quantified with Qubit dsDNA HS Assay; Labeled DNA CV = Coefficient of Variation (CV = standard deviation/mean) from the two readings; Avg N50 (≥150kpb) = N50 of DNA molecules that are 150kbp or longer; Avg N50 (≥20kpb) = N50 of the molecules that are 20kbp or longer; Avg Label Density = Average number of labels per 100 kbp for the molecules that are 150kbp or longer; Avg Map Rate = Percentage of molecules that are 150kbp or longer mapped to the reference; Effective Coverage = Total amount of aligned DNA divided by the size of the reference genome times the map rate; Avg PLV = Percentage of labels absent in reference; Avg NLV = Percentage of reference labels absent in molecules; DNA collected = Total amount of DNA from molecules that are 150kbp or longer. ST = Standard Deviation

Table S1: Optical Genome Mapping Quality Parameters.

	Rare variant pipeline			De novo assembly		
**Patient**	**SV**	**VAF**	**Confidence**	**SV**	**VAF**	**Confidence**
**1**	No relevant SVs detected	-	-	No relevant SVs detected	-	-
**2**	No relevant SVs detected	-	-	No relevant SVs detected	-	-
**3**	No relevant SVs detected	-	-	No relevant SVs detected	-	-
**4**	1q31.3q44(194830658_248943333)x3	10%	0.88	1q21.2q44(148647980_248943333)x3	10%	0.88
	6p25.3p22.1(76216_27868248)x1	10%	0.76	6p25.3p22.3(76216_19002568)x1	14%	1
	t(12;17)(q24.31;p13.1)	19%	0.99	t(12;17)(q24.31;p13.1)	11%	0.61
	Xq11.1q28 (63301777_155792271)x1	10%	0.41	Xq11.1q28 (62521449_156025612)x1	10%	0.73
**5**	1q21.1q44(145439805_248943333)x3	33%	1	1q21.1q44(144234187_248943333)x3	31%	1
	7q11.21q36.3(62332055_159334984)x1	31%	1	7q11.21 q36.3 (62332055_157634263)x1	31%	1
	(9)x3	30%	1	(9)x3	30%	1
**6**	4q24(105025784_105410120)x1	6%	0.99	No relevant SVs detected	-	-
**7**	No relevant SVs detected	-	-	No relevant SVs detected	-	-
**8**	No relevant SVs detected	-	-	No relevant SVs detected	-	-
**9**	No relevant SVs detected	-	-	9p24.2p13.3(0_ 35495657)x2 hmz	-	-
**10**	t(2;11)(q37.1;q23.2)	54%	0.98	t(2;11)(q37.1;q23.2)	40%	0.6
	7p21.1p14.3(20628867_30992020)x1	49%	1	7p21.1p14.3(20628867_31009519)x1	47%	1
	t(7;11)(q31.31;q24.1)	47%	0.97	t(7;11)(q31.31;q24.1)	28%	0.3
	7q31.31q32.1 (118375331_129537957)x1	43%	0.99	7q31.31q32.1 (118375331_129537957)x1	48%	1
	(8)x3	49%	1	(8)x3	43%	1
	11q23.2q23.3(113532792_115861804)x1	45%	1	11q23.2q24.1(113528118_122641085)x1	46%	1
**11**	7q34q35(142383192_144044869)x1	34%	1	7q34q35(142359655_144082533)x1	37%	1
	t(7;13)(q34;q14.2)	40%	1	t(7;13)(q34;q14.2)	36%	0.39
	13q14.13q14.2(45449589_48811560)x1	39%	1	13q14.13q14.2(45284560_48793119)x1	37%	1
	20q11.21q11.22(32412577_34753168)x1	38%	1	20q11.21q11.22(32412577_34729749)x1	36%	1
**12**	No relevant SVs detected	-	-	No relevant SVs detected	-	-
**13**	No relevant SVs detected	-	-	No relevant SVs detected	-	-
**14**	No relevant SVs detected	-	-	No relevant SVs detected	-	-
**15**	No relevant SVs detected	-	-	No relevant SVs detected	-	-

Table S2: Comparison between rare variant analysis y and the *de novo* assembly finding

Patient	Rare variant pipeline			*De novo* assembly		
	**SV**	**VAF**	**Confidence**	**SV**	**VAF**	**Confidence**
**16**	2p23.3p23.2(27431781_28419901)x1	35%	1	2p23.3p23.2(27431781_28418674)x1	35%	1
	14q32.12q32.31 (92912843_102275947)x1	29%	1	14q32.2q32.31(100650415_102282650)x1	31%	1
	t(2;14)(p23.2;q32.12)	42%	0.99	t(2;14)(p23.2;q32.12)	27%	0.04
**17**	No relevant SVs detected	-	-	No relevant SVs detected	-	-
**18**	No relevant SVs detected	-	-	No relevant SVs detected	-	-
**19**	(9)x3	9%	0.1	(9)x3	7%	0.84
	20q11.21q13.32(31688875_58520469)x1	18%	1	20q11.21q13.32(31971356_58555654)x1	17%	1
**20**	No relevant SVs detected	-	-	No relevant SVs detected	-	-
**21**	t(1;12)(p35.2;q13.13)	5%	0.22	No relevant SVs detected	-	-
	t(1;14)(p35.2;q32.31)	14%	0.98	No relevant SVs detected	-	-

Table S2: *Cont.*

Patient	OGM new findings	Validation technique	Validation result	Interpretation
**4**	1q21.2q23.2x3,<br>t(12;17)(q24.31;p13.1)	FISH<br>ZytoLight ® SPEC MEF2D/BCL9 TriCheck™<br>Metasystems XCP12 green and XCP17 orange	nuc ish(MEF2Dx3,BCL9x3)[15/100]<br>46,XX.ish t(12;17)(q24.31;p13.1) (wcp12+,wcp17-)	Confirmed
**5**	1q21.1q44x3,<br>7q11.21q36.3x1<br>(9)x3	SNP array	arr[GRCh38] 1q21.3(151945023_248808397)x3<br>arr[GRCh38] 7q11.21 q35 (66023357_146394056)x1<br>arr[GRCh38] (9)x3	Confirmed
**6**	4q24x1	FISH<br>Metasystems XL TET2 locus-specific probe	nuc ish (TET2x1)[10/100]	Confirmed
**9**	9p24.2p13.3x2 hmz	SNP array	arr[GRCh38] 9p24.3p21.1(216,124_33,190,241)x2 hmz	Confirmed
**10**	t(2;11)(q37.1;q23.2)<br>t(7;11)(q31.31;q24.1)<br>7q31.31q32.1x1<br>11q23.2q24.1x1	FISH<br>Metasystems XCP2 green and XCP11 orange<br>Metasystems XCP7 orange and XCP11 green<br>+<br>SNP array	46,XY. ish t(2;11)(q37.1;q23.2)) (wcp2+,wcp11-)<br>46,XY. ish t(7;11)(q31.31;q24.1) (wcp7+,wcp11-)<br>arr[GRCh38] 7q31.31q32.1(120,408,852_129,549,635)x1<br>arr[GRCh38] 11q23.2q24.1(113,579,228_122,624,689)x1	Confirmed
**11**	7q34q35x1<br>13q14.13q14.2x1<br>20q11.21q11.22x	SNP array	arr[GRCh38] 7q34q35 (1142359655_144082533)<br>arr[GRCh38] 13q14.13q14.2x1(45284560_48793119)<br>arr[GRCh38] 20q11.21q11.22x1(32412577_34729749)	Confirmed
**16**	2p23.3p23.2x1<br>14q32.12q32.31x1<br>t(2;14)(p23.2;q32.12)	FISH<br>Metasystems XCP2 green and XCP14 orange<br>+<br>SNP array	arr[GRCh38] 2p23.3p23.2(27,533,582_28,419,761)x1<br>arr[GRCh38] 14q32.2q32.31(100,713,259_102,249,264)x1	t(2;14) not confirmed by FISH<br>Losses in chromosomes 2 and 14 confirmed by SNP array
**19**	20q11.21q13.32x1	FISH	nuc ish<br>(D8Z1x2,D20S108/D20S858x1,RH74808/RH67654x2)[20/100]	Confirmed

Table S3: Validation of results.

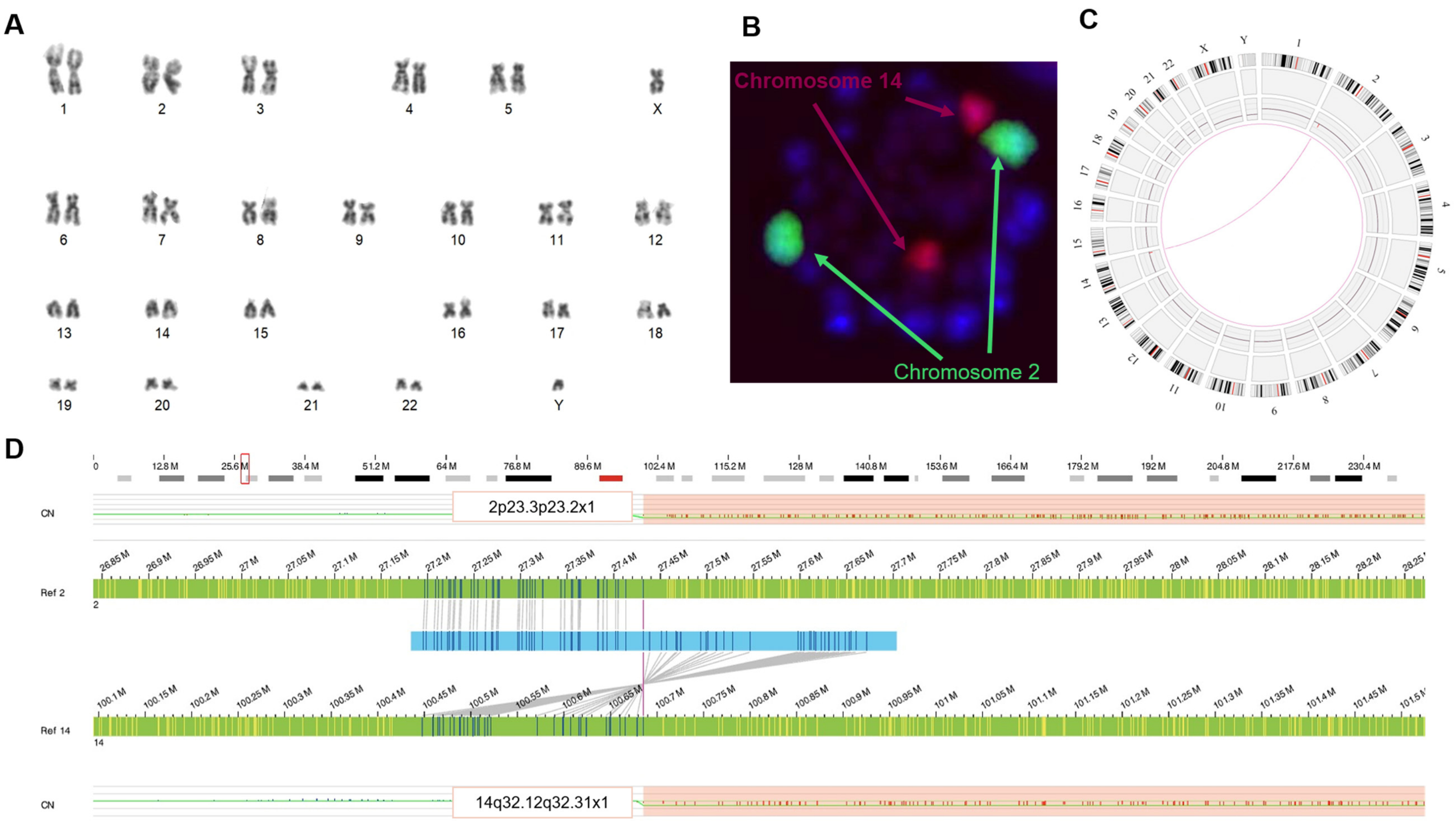

Figure S1: Patient #16 and discordance between CBA & FISH vs OGM. (a) CBA reveals a 46,XY [20] karyotype. (b) Metaphase FISH with whole chromosome painting failed to detect t(2;14)(p23.2;q32.12). (c) Circos plot showing the translocation t(2;14)(p23.2;q32.12) with material losses in adjacent regions. (d). Detail of the translocation detected by OGM.
